# A Synergistic Social Work–Ethnic Education Intervention for Reducing Dropout Risk Among Male Students in Central Guangxi Zhuang Vocational High Schools: A Mixed-Methods and Quasi-Experimental Study

**DOI:** 10.3390/bs16061023

**Published:** 2026-06-18

**Authors:** Guobin Huang, Lu Hai

**Affiliations:** 1 Social Work Direction of the School of Health Sciences, City University of Macau, Macau, China; w25091105284@cityu.edu.mo; 2 School of Education, Minzu University of China, Beijing 100081, China

**Keywords:** dropout risk, vocational education, school social work, school belonging, academic self-efficacy

## Abstract

This study evaluated a synergistic intervention integrating school social work and ethnic education for reducing dropout-related risk among male students in Zhuang vocational secondary schools in central Guangxi, China. Using a quasi-experimental mixed-methods design with baseline, post-intervention, and follow-up assessments, 457 students were enrolled and 435 were included in the final analysis. Compared with usual support, the intervention group showed a larger reduction in the dropout risk index at follow-up, β = −0.37, SE = 0.08, 95% CI [−0.52, −0.22], *p* < 0.001, and a lower likelihood of chronic absenteeism, OR = 0.56, 95% CI [0.34, 0.91], *p* = 0.020. The retention difference was positive but less precise, OR = 1.70, 95% CI [0.79, 3.67], *p* = 0.174. The intervention group also reported higher school belonging, β = 0.33, SE = 0.06, *p* < 0.001, and academic self-efficacy, β = 0.30, SE = 0.06, *p* < 0.001. Parallel mediation analysis suggested that these two protective factors accounted for part of the intervention-associated difference in dropout risk, with a total indirect effect of −0.20, 95% CI [−0.28, −0.12], *p* < 0.001. The findings suggest that culturally responsive practices, when combined with tiered case management and family engagement, may help strengthen protective processes and slow the accumulation of dropout-related risks. This study provides context-sensitive evidence for designing school retention interventions in vocational schools serving ethnic minority communities, while the quasi-experimental design warrants cautious interpretation.

## 1. Introduction

In recent years, vocational education has been increasingly positioned in many countries as an important policy instrument for addressing youth unemployment, strengthening skills development, and supporting social mobility (Neuenschwander et al., 2024), yet dropout and chronic absenteeism continue to undermine training quality, educational continuity, and equity within vocational pathways (Wilson et al., 2011). In China, the stability of secondary vocational education is shaped not only by school-based factors, but also by family decision-making, social perceptions of vocational tracks, expectations for further education, and the pull of local labor markets (Korpershoek et al., 2020). Existing school-based responses have generally centered on early warning and tiered support, identifying high-risk students through attendance, discipline, and academic indicators, and then responding through homeroom teacher follow-up, home-school communication, and psychological support. In parallel, brief behaviorally informed strategies, such as attendance feedback, reminder systems, and parent-directed normative prompts, have been used because they are comparatively low-cost and scalable; however, these approaches often appear more effective for short-term attendance management than for sustained school retention (Fenizia & Parrello, 2025; Hong et al., 2021).

This limitation becomes more visible when attention shifts to male students in vocational high schools, particularly those studying in ethnic minority regions. In these settings, dropout is rarely the product of a single academic problem. More often, it reflects an accumulated risk process involving repeated absenteeism, peer influence, strained classroom experiences, preference for short-term earnings over delayed educational returns, fragile family support, and uncertainty about future pathways (Sulimani-Aidan & Melkman, 2022). For this reason, interventions that rely only on attendance alerts or one-off communication may help suppress temporary fluctuations in absenteeism, yet remain insufficient for stabilizing long-term school commitment. Their effectiveness also tends to vary across schools, classes, and students with different baseline risk profiles, suggesting that contextual fit and mechanism specificity matter. Among students in ethnic minority areas, differences in linguistic resources, cultural experience, and identity recognition may further shape how school norms are interpreted, how support is received, and whether participation can be sustained over time (Jiang et al., 2022).

Current intervention research and practice remain insufficient in at least three respects. First, many dropout prevention models are built on general risk indicators but pay limited attention to cultural differences, identity formation, and language-related adaptation in ethnic minority settings. As a result, support may remain administratively present but experientially distant, producing compliance rather than meaningful engagement (Dockery, 2012). Second, many school responses remain concentrated at the level of classroom management or psychological guidance, without adequately linking student support to family systems and community resources. This weakens the capacity of interventions to address structural pressures such as labor migration, caregiver strain, and fragmented supervision beyond school. Third, although some programs improve short-term process indicators, including attendance during specific intervention periods or immediate participation rates, evidence remains less clear on whether they can produce durable reductions in dropout-related risk. At the same time, existing studies often provide limited explanation of underlying mechanisms, particularly regarding why an intervention works, for whom it works best, and under what contextual conditions its effects are more likely to be sustained (Wilson & Tanner-Smith, 2013). These gaps are especially important in vocational education, where students’ educational decisions are closely tied to perceived returns, family realities, and visible career prospects. Without strengthening school belonging, academic self-efficacy, and the perceived meaning of continued learning at the same time, intervention effects may be difficult to maintain (World Bank Group, 2018).

The present study responds to these gaps by examining a collaborative intervention that integrates school social work with ethnic education in vocational schools serving Zhuang communities in central Guangxi. Conceptually, this intervention is not treated as a simple juxtaposition of two components. Rather, it is designed as a coordinated support model in which social work addresses risk accumulation through case management, family engagement, and continuous follow-up, while ethnic education contributes culturally responsive pedagogy, identity affirmation, and stronger connections between learning, local culture, and future development (Szabó et al., 2024). The intervention therefore seeks to connect individual support, family participation, and school-level meaning construction within a shared collaborative framework. At the individual level, high-risk male students receive learning support, emotional and conflict management, peer relationship adjustment, and career-planning guidance through ongoing case services. At the family level, home visits, caregiver communication, and resource linkage are used to strengthen guardians’ involvement and practical support capacity. At the school level, ethnic education is repositioned from activity-based delivery toward culturally responsive pedagogy that links Zhuang cultural resources with vocational learning and future-oriented skill development. A regular collaboration mechanism among social workers, homeroom teachers, and ethnic education instructors is then used to support information sharing, joint assessment, and coordinated response.

This study therefore addresses a more specific question than whether “combined support” is broadly useful. It asks whether a culturally situated, school-based collaborative intervention can reduce dropout-related risk among male vocational students in an ethnic minority context, and whether its effects are associated with changes in key protective processes, especially school belonging and academic self-efficacy. On this basis, the study has three objectives: first, to evaluate whether the intervention is associated with lower dropout risk, lower chronic absenteeism, and higher retention relative to usual support; second, to examine whether school belonging and academic self-efficacy function as explanatory pathways linking intervention exposure to student outcomes; and third, to generate practice-oriented evidence for more context-sensitive school retention governance in vocational education in ethnic regions. By doing so, the study seeks to contribute not only an applied intervention model, but also a more mechanism-focused account of how culturally responsive and relational forms of support may operate in vocational education settings marked by ethnic diversity and dropout vulnerability.

## 2. Conceptual Framework and Hypotheses

### 2.1. Theoretical Foundations

This study is grounded in a layered explanatory framework for understanding dropout-related risk among male vocational high school students in ethnic minority regions. Rather than treating dropout as a single event, the present study conceptualizes it as the outcome of a cumulative process shaped by the interaction of individual, family, school, and broader social conditions (Peguero & Shaffer, 2015). Within this framework, the proposed intervention integrates school social work and ethnic education as two coordinated, rather than merely coexisting, sources of support.

First, drawing upon Bronfenbrenner’s bioecological systems theory (Gubbels et al., 2019), dropout-related risk is conceptualized not as an isolated individual deficit, but as an accumulated vulnerability emerging from the reciprocal interactions across micro-, meso-, and exo-systems (e.g., family structures, classroom climates, and peer networks). For male students in vocational education, learning frustration, classroom maladjustment, peer influence, weak family supervision, economic pressure, and early labor market attraction may interact over time, producing repeated absenteeism, disengagement from school life, and eventually withdrawal or prolonged detachment (Cents-Boonstra et al., 2019). This perspective is especially relevant in ethnic minority settings, where linguistic experience, cultural identity, and institutional fit may further shape how students interpret school expectations and respond to support (Allen et al., 2018).

Second, the risk–protection framework helps identify where intervention can realistically act on this process. In the present context, major risk factors include chronic absenteeism, disciplinary conflict, learning difficulty, low school belonging, low academic self-efficacy, fragile family support, and accumulated exposure to disengaging peer norms. Protective factors include stable attendance routines, supportive family involvement, positive peer relationships, clearer future goals, stronger school attachment, and greater confidence in managing academic and vocational tasks (Andersen et al., 2018). This framework is important because it shifts the focus from dropout as a final outcome to dropout-related risk as a modifiable process. In other words, intervention becomes meaningful not only when it prevents formal withdrawal, but also when it interrupts the pathways through which disengagement intensifies.

Third, the present study uses school belonging and academic self-efficacy as the two central mediating variables linking intervention exposure to student outcomes. School belonging captures whether students feel accepted, respected, and meaningfully connected to the school environment. Academic self-efficacy reflects students’ confidence in their ability to complete learning tasks, cope with difficulty, and persist in vocational training (Böhn & Deutscher, 2022), a psychological state that is fundamentally mediated by the interaction between a student’s individual agency and the specific affordances of the vocational learning environment (Billett, 2014). These two mechanisms are especially relevant in this study because they connect the two components of the intervention. Ethnic education contributes by strengthening meaning construction, identity recognition, and cultural responsiveness, thereby reinforcing students’ sense of connection to school. School social work contributes by providing case management, family communication, emotional support, and problem-solving guidance, thereby improving coping capacity and efficacy beliefs (Korpershoek et al., 2020). When these two forms of support operate together, they are expected to strengthen protective processes more effectively than routine support alone.

Taken together, the theoretical logic of this study is that dropout-related risk among male vocational students in ethnic minority regions is produced through cumulative ecological pressures, can be interpreted through a risk–protection lens, and may be reduced when collaborative intervention strengthens school belonging and academic self-efficacy as key protective mechanisms. This integrated framework provides the conceptual basis for the hypotheses tested in the present study.

### 2.2. Key Constructs and Operational Orientation

The central outcome domain of this study is dropout-related risk. In this study, dropout-related risk refers not only to formal school withdrawal, but also to a patterned tendency toward educational disengagement that may be reflected in chronic absenteeism, extended absence, transfer associated with clear disengagement signals, and elevated composite risk indicators derived from administrative and survey-based information. Because formal dropout is a relatively late-stage event, the study places greater analytical emphasis on risk-related outcomes that can be observed earlier and more reliably within the school process (Neuenschwander et al., 2024).

Accordingly, three outcome variables are emphasized in the empirical model: dropout risk, chronic absenteeism, and retention. Dropout risk is treated as a composite indicator reflecting accumulated warning signs across attendance, discipline, academic functioning, and self-reported withdrawal tendency. Chronic absenteeism refers to sustained absence above the school’s warning threshold during the observation window. Retention refers to continued enrollment without school withdrawal or transfer out during the study period. Together, these outcomes allow the study to capture both proximal and more consequential forms of dropout-related vulnerability.

The core independent variable is exposure to the synergistic intervention. At the most basic level, this refers to whether students were assigned to the intervention condition or the comparison condition. At the implementation level, intervention exposure also includes the practical dosage of support, such as participation in social work case services, family engagement activities, group-based support sessions, and ethnic education activities delivered within the collaborative framework. However, because the main analytical design is based on group comparison under a quasi-experimental structure, intervention status remains the primary independent variable in the formal hypothesis system.

The two principal mechanism variables are school belonging and academic self-efficacy. School belonging refers to students’ perceived acceptance, recognition, and emotional connection within the school environment. Academic self-efficacy refers to students’ confidence in their ability to manage academic and vocational learning tasks, persist through difficulty, and achieve expected learning goals. These two constructs are treated as parallel mediators because they represent distinct but related pathways through which the intervention may influence dropout-related outcomes.

Other contextual factors, including family support, guardian involvement, peer influence, left-behind experience, academic baseline, and economic pressure, are treated as background or control conditions rather than central mediators in the main model. Although these factors may shape dropout-related risk and may condition intervention responsiveness, they are not positioned as primary explanatory mechanisms in the present hypothesis structure. This distinction is important for maintaining coherence between the conceptual framework, the stated hypotheses, and the empirical estimation strategy used later in the study.

### 2.3. Mechanisms of the Synergistic Intervention

**Figure 1:**
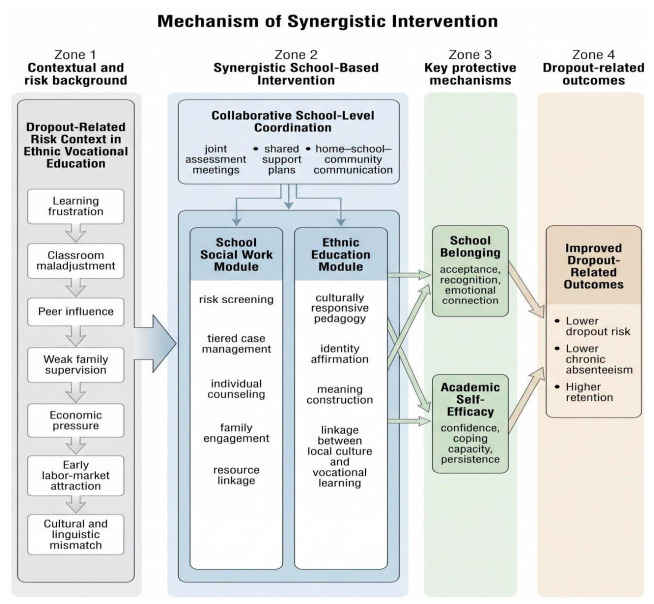
Mechanism of Synergistic Intervention.

As illustrated in the conceptual model (Figure 1), the synergistic intervention operationalizes the risk-protection framework by mapping specific support modules onto the ecological risk zones (Zone 1). Specifically, the dual-module structure within the synergistic school-based intervention (Zone 2) combines school social work and ethnic education to activate key protective mechanisms (Zone 3), ultimately aiming to improve dropout-related outcomes (Zone 4). Its core assumption is that dropout-related risk in ethnic vocational settings cannot be effectively addressed through isolated school management measures alone. Instead, intervention must simultaneously respond to relational disconnection, weak efficacy beliefs, family strain, and the mismatch between school learning and students’ cultural or future-oriented meaning systems.

The first component is the school social work module. This module uses risk screening and tiered case management as its operational entry points. For students showing higher levels of risk, it provides continuous follow-up, individual counseling, emotional and conflict management support, peer relationship adjustment, and problem-solving guidance. At the family level, social work intervention extends to caregiver communication, home visits, and resource linkage, with the aim of improving supervision quality, reducing family–school disconnection, and addressing practical pressures that may intensify absenteeism or withdrawal intentions. In this sense, the social work component is designed primarily to stabilize support relationships and strengthen students’ capacity to cope with difficulty.

The second component is the ethnic education module. This module adopts a culturally responsive orientation by linking ethnic cultural resources, identity affirmation, and vocational learning, effectively moving toward a “culturally sustaining” framework that validates and fosters linguistic and cultural pluralism as a prerequisite for academic persistence (Paris & Alim, 2017). Rather than treating ethnic education as an isolated cultural activity, the intervention repositions it as a school-based mechanism for enhancing the perceived meaning of learning, improving students’ sense of recognition within the institution, and reducing alienation associated with linguistic or cultural mismatch. Through this pathway, ethnic education is expected to contribute especially to school belonging, while also helping students perceive continued learning as more personally and socially meaningful.

The synergy between the two modules lies not simply in simultaneous delivery, but in structured coordination. Joint assessment meetings, shared support plans, and home–school–community communication create a closed-loop system in which information is exchanged, priorities are reviewed, and intervention strategies are adjusted over time. Through this collaborative mechanism, improvements in relational support, meaning construction, and coping confidence are expected to translate into better attendance, lower dropout-related risk, and more stable retention. In short, the intervention is theorized to work because it addresses both the social conditions and the motivational processes that sustain disengagement.

### 2.4. Hypotheses Development

Based on the theoretical framework outlined above, this study develops a set of hypotheses linking intervention exposure, mechanism variables, and dropout-related outcomes. The synergistic intervention is expected to improve student outcomes by combining tiered social work support, family engagement, and culturally responsive ethnic education. Because the study adopts a quasi-experimental design, the hypotheses are expressed in terms of expected associations and differences between conditions, rather than strong causal claims.

First, at the outcome level, students exposed to the synergistic intervention are expected to show lower dropout-related risk and chronic absenteeism, and higher retention, than students receiving usual support. This expectation follows from empirical evidence that multi-tiered collaborative interventions can effectively interrupt the accumulation of risk across school, family, and motivational domains, thereby enhancing school retention (Gubbels et al., 2019; Tanner-Smith & Wilson, 2013).

Second, at the mechanism level, the intervention is expected to be associated with higher school belonging and higher academic self-efficacy. This mechanism is grounded in recent literature demonstrating that culturally responsive pedagogy significantly strengthens minoritized students’ sense of acceptance and school belonging (Korpershoek et al., 2020), while structured psychosocial support bolsters academic self-efficacy by equipping students with effective coping strategies (Böhn & Deutscher, 2022).

Third, school belonging and academic self-efficacy are expected to be associated with more favorable dropout-related outcomes. Students who feel connected to school and believe they can successfully manage academic and vocational demands are less likely to disengage, avoid attendance, or move toward withdrawal.

Finally, the study proposes that school belonging and academic self-efficacy function as parallel explanatory pathways linking intervention exposure to dropout-related outcomes. In other words, the intervention is expected to be associated with outcome differences partly because it strengthens these two protective mechanisms.

**H1.**  *Synergistic intervention exposure is negatively associated with dropout risk and chronic absenteeism and positively associated with retention.*

**H2.**  *Synergistic intervention exposure is positively associated with school belonging.*

**H3.**  *Synergistic intervention exposure is positively associated with academic self-efficacy.*

**H4.**  *School belonging is negatively associated with dropout risk and chronic absenteeism and positively associated with retention.*

**H5.**  *Academic self-efficacy is negatively associated with dropout risk and chronic absenteeism and positively associated with retention.*

**H6.**  *School belonging and academic self-efficacy mediate the relationship between synergistic intervention exposure and dropout-related outcomes.*

### 2.5. Analytical Model Specification

**Figure 2:**
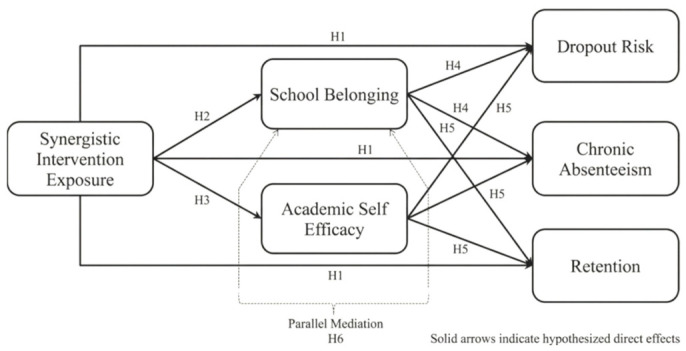
Analytical Model of Dropout Risk Reduction.

Figure 2 presents the analytical model used in this study. The model is structured around one core independent variable, two parallel mediators, and three dropout-related outcomes. Synergistic intervention exposure functions as the independent variable. School belonging and academic self-efficacy are specified as parallel mediators. Dropout risk, chronic absenteeism, and retention are treated as the outcome variables.

The model contains three connected layers of estimation. The first layer examines whether intervention exposure is associated with overall between-group differences in dropout-related outcomes. The second layer evaluates whether intervention exposure is associated with variation in school belonging and academic self-efficacy. The third layer tests whether these mechanism variables are associated with dropout-related outcomes and whether indirect pathways can be identified through parallel mediation analysis. In this way, the model is designed to examine both whether the intervention is associated with better student outcomes and how those associations may be partly transmitted through key protective processes.

Because the study uses a quasi-experimental design rather than randomized assignment, the analytical strategy emphasizes careful estimation of between-group differences, adjustment for baseline conditions and clustering structure, and cautious interpretation of mediation pathways. Separate models are fitted for each outcome, and direct, indirect, and total associations are interpreted in a way that remains consistent with the design and the level of inference supported by the data. This analytical specification therefore links the conceptual framework to the empirical strategy in a coherent and testable manner.

## 3. Methods

### 3.1. Study Design

This study utilized a three-wave, quasi-experimental mixed-methods design to evaluate a synergistic social work–ethnic education intervention targeting dropout risk among male Zhuang vocational students. The design prioritized ecological validity while incorporating rigorous covariate controls and clustering adjustments to mitigate potential selection bias. The quantitative component used a three-wave design with baseline (T0), post-intervention (T1), and follow-up (T2) assessments, together with a comparison group receiving usual school support. Because individual randomization was not feasible within the participating schools, allocation was conducted at the class level under real school conditions. This design strengthened ecological validity but also introduced potential selection bias; therefore, the analysis incorporated baseline equivalence checks, adjustment for observed baseline covariates, and class-level clustering control to reduce, although not eliminate, threats to internal validity.

The qualitative component was embedded within the overall design to support interpretation of the quantitative results rather than to provide an independently weighted qualitative trial strand. Semi-structured interviews and process documentation were used to clarify how the intervention was implemented, how participants experienced it, and why some effects may have been stronger or weaker across student groups and school contexts. To achieve meaningful integration, qualitative materials were utilized during the interpretation stage to contextualize the quantitative variations observed in school belonging, academic self-efficacy, and subgroup responsiveness. This mixed-methods structure was therefore intended to address two related questions: whether the intervention was associated with more favorable dropout-related outcomes, and how these patterns could be understood in light of school processes, family engagement, and culturally responsive implementation.

At baseline, the research team collected administrative information on attendance, disciplinary records, and academic performance, together with student survey data on dropout-related risk and psychosocial indicators. Students were then allocated by class, or by grade-level class cluster where required by school scheduling conditions, to either the intervention condition or the comparison condition. The intervention group received the collaborative program consisting of social work risk screening, case management, family engagement, culturally responsive ethnic education activities, and joint school-level coordination. The comparison group continued to receive routine school management, standard moral education, and existing pastoral or disciplinary support, but did not receive the structured collaborative intervention package described in this study. No individual student was reassigned after baseline allocation, and all students were analyzed according to their original allocation in the main analysis to preserve the intention-to-treat logic of the study.

The design incorporated three formal measurement points. T0 represented the pre-intervention baseline; T1 captured the immediate post-intervention assessment; and T2 was used to examine short-term maintenance, including retention and follow-up attendance-related outcomes. The primary outcomes were dropout risk, chronic absenteeism, and retention. Administrative indicators, including absence days, tardiness, and disciplinary events, were included to strengthen behavioral measurement and reduce sole reliance on self-report data. School belonging and academic self-efficacy were specified as the two principal mechanism variables in line with the conceptual model. The three-wave structure also allowed the study to distinguish baseline comparability, immediate post-intervention change, and short-term maintenance, although the follow-up period remained insufficient for making claims about long-term educational trajectories.

To strengthen methodological transparency, the study also included process evaluation and implementation monitoring. These procedures documented intervention coverage, actual service delivery, participation intensity, and fidelity to the planned collaborative model. Standardized records were maintained for joint assessment meetings, shared support plans, and service completion, allowing the research team to interpret variation in outcomes with reference to implementation quality rather than outcome data alone. During the middle and later phases of the intervention, qualitative interviews were conducted with students, guardians, homeroom teachers, ethnic education instructors, and school social workers across different levels of risk exposure and intervention participation. These materials were used to triangulate the quantitative findings and to clarify how dropout motivation, school connection, family dynamics, and cultural responsiveness interacted during implementation.

**Figure 3:**
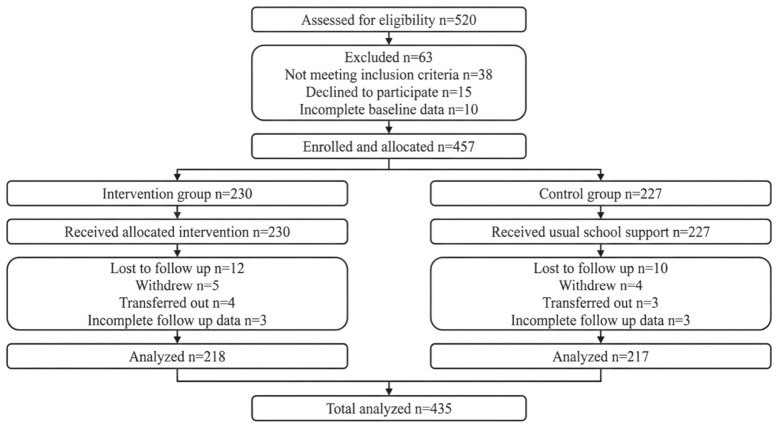
Study Flowchart.

Overall, the design prioritized ecological validity while preserving as much analytical rigor as possible under school-based constraints. Rather than claiming experimental proof, the study was designed to generate context-sensitive evidence on intervention-associated outcome differences, plausible mechanism pathways, and implementation feasibility in vocational schools serving ethnic minority communities. Accordingly, all findings are interpreted as adjusted associations within a quasi-experimental school-based design, not as definitive causal effects. The study flow is presented in Figure 3.

### 3.2. Setting and Participants

The study was conducted in three public secondary vocational schools located in county-level and suburban areas of the central Guangxi Zhuang Autonomous Region, China. These schools offered major programs in intelligent manufacturing, electromechanical technology application, automotive maintenance, computer application, and tourism services and management. The target population consisted of male students enrolled in Grades 10 to 12. The focus on male students reflected the study’s specific concern with a subgroup that, in the participating schools, showed recurrent patterns of absenteeism, disengagement, and dropout-related vulnerability within the vocational education context.

Students were eligible for inclusion if they met all of the following criteria: (1) they were formally enrolled and in active academic status at the participating schools; (2) they completed the baseline survey and provided informed consent, with guardian consent obtained where required; and (3) complete baseline administrative records were available for attendance, discipline, and academic performance. Students were excluded if they had severe cognitive or communication difficulties preventing valid participation in the assessments, if key baseline data were missing and could not be verified from school records, or if they were already in long-term non-attending or de facto withdrawn status before the intervention period began. These criteria were applied before group allocation to reduce the risk that post-allocation exclusions would distort group comparability.

To reduce contamination between conditions, allocation was conducted at the class level within each school wherever feasible. Classes assigned to the intervention condition received the full collaborative intervention package, whereas classes assigned to the comparison condition continued under standard school management and routine moral education arrangements. Class-level allocation was selected because the intervention involved group activities, teacher–social worker coordination, and shared classroom routines that could not be realistically delivered to selected individuals within the same class without spillover. At baseline, 457 students were enrolled, including 230 in the intervention group and 227 in the comparison group. By the end of follow-up, 435 students remained in the analytic sample, comprising 218 in the intervention group and 217 in the comparison group. Attrition was primarily related to transfer, withdrawal, or incomplete follow-up data rather than intervention refusal. The attrition rate was 5.2% in the intervention group and 4.4% in the comparison group, suggesting no substantial imbalance in follow-up loss between conditions.

Baseline equivalence between groups was examined using descriptive and inferential comparisons across demographic, academic, and risk-related indicators. As shown in Table 1, no statistically significant between-group differences were observed at baseline on age, grade, ethnicity, boarding status, left-behind experience, baseline absence burden, chronic absenteeism, academic score, disciplinary history, or perceived family economic pressure. For continuous normally distributed variables, independent-sample *t* tests were used; for skewed absence data, non-parametric tests were applied; and for categorical variables, chi-square tests were used. These baseline comparisons do not rule out unmeasured selection bias, but they indicate that the two groups were broadly comparable on the main observed baseline characteristics included in the study. These variables were also considered in adjusted analyses to further reduce confounding from observed pre-intervention differences.

### 3.3. Sampling Strategy and Sample Size

The study used a cluster-based sampling and allocation strategy in which schools served as implementation settings and classes served as the primary units for participant grouping. This approach was adopted for both practical and methodological reasons. Practically, the intervention was delivered through class-based school routines and collaborative staff structures; methodologically, class-level grouping reduced spillover between intervention and comparison students within the same school day environment. To improve coverage across the participating schools, students from multiple grades and program areas were included. Because students were nested within classes and schools, the sampling structure was explicitly considered during analysis through class-level random effects or clustered standard errors, depending on model specification and convergence.

Sample size was determined on the basis of two considerations. First, the study sought to retain sufficient statistical information to detect small-to-moderate between-group differences in dropout-related outcomes, especially dropout risk and chronic absenteeism, under a clustered school-based design. Second, the baseline enrollment target was set high enough to accommodate expected attrition during the follow-up period. A total of 457 students were enrolled at baseline, and 435 were retained in the final analytic sample, corresponding to an overall attrition rate of 4.8%. This retention level was considered acceptable for the planned longitudinal analyses and mediation models. Although no individual-level random assignment or formal randomized power calculation was possible under the school-based implementation conditions, the final analytic sample provided adequate information for estimating adjusted group-by-time associations, mediation pathways, and exploratory subgroup patterns.

Because the intervention was allocated by class rather than by individual randomization, subsequent analyses accounted for clustering at the class level. The sample size was therefore judged in relation not only to the number of students, but also to the need for stable estimation under mixed-effects modeling and clustered standard-error procedures. The primary inferential emphasis was placed on adjusted longitudinal estimates rather than unadjusted endpoint differences. Sensitivity analyses were planned to examine whether the main findings were robust after accounting for baseline covariates, incomplete follow-up data, and clustering structure. This approach was used to strengthen analytical rigor while remaining consistent with the quasi-experimental nature of the study.

### 3.4. Measures and Data Sources

The measurement framework combined administrative data with student-reported survey data. Administrative data were used to capture observable behavioral and school-status outcomes, whereas survey measures were used to assess psychosocial mechanisms and background characteristics. This dual-source design was intended to reduce common-method bias and improve the construct validity of the main outcome and mechanism indicators. Measurements were collected at T0, T1, and T2, except for baseline covariates, which were recorded at T0 only, shown as Table 2.

The primary outcome, dropout risk, was operationalized as a composite risk index integrating four domains: attendance burden, disciplinary risk, academic warning signals, and self-reported withdrawal tendency. The index was constructed before the main outcome analysis according to a predefined scoring rule. Each domain was first transformed into a standardized z-score within the analytic sample, with higher values consistently indicating greater dropout-related vulnerability. The four standardized components were then combined using equal weights, with each domain contributing 25% to the final composite score. Equal weighting was selected because the study aimed to capture a broad risk profile rather than privilege one administrative indicator over another, and because no externally validated weighting system was available for this specific ethnic vocational school context. Attendance burden included absence days and tardiness frequency; disciplinary risk included any disciplinary incident and repeated behavioral warning; academic warning signals were derived from low or declining academic performance relative to school expectations; and self-reported withdrawal tendency was measured through student responses indicating intention to disengage, leave school, or interrupt enrollment. The final composite index was treated as a continuous standardized score, where positive values indicated higher-than-average dropout-related risk and negative values indicated lower-than-average risk. Internal coherence and distributional properties of the index, including inter-domain correlations, scale dispersion, and sensitivity to alternative weighting specifications, were examined and are reported in Section 4. This composite outcome was used because formal dropout alone is a relatively infrequent late-stage event and may not fully capture meaningful changes in dropout-related vulnerability during the study window.

Chronic absenteeism was defined as an absence proportion exceeding the school’s warning threshold during the relevant observation period. In the participating schools, this threshold corresponded to repeated absence reaching or exceeding the administrative warning level used by student affairs offices during the semester monitoring cycle. Students meeting this threshold were coded as 1, and those below the threshold were coded as 0. Retention was defined as continued enrollment without formal withdrawal or transfer out during the follow-up period. Attendance indicators, disciplinary incidents, and enrollment status were extracted directly from school systems or student affairs records. Administrative variables were checked against class attendance logs and student status registries to reduce recording error.

School belonging was measured using a school connectedness item set in which higher mean scores indicated stronger perceived acceptance, recognition, and emotional connection to school. The item set was adapted from commonly used school belonging and school connectedness measures and revised for the vocational school context. Items asked students to rate whether they felt accepted by teachers and classmates, whether they felt recognized within school life, and whether school participation was meaningful to them. Responses were recorded on a five-point Likert-type scale ranging from 1 = strongly disagree to 5 = strongly agree. The final score was calculated as the mean of all valid items, with higher scores indicating stronger school belonging.

Academic self-efficacy was measured using student-reported items assessing confidence in managing learning tasks, mastering vocational content, and persisting through academic difficulty. The measure was adapted from established academic self-efficacy instruments and contextualized for vocational learning tasks, including confidence in completing coursework, understanding professional training content, and persisting when learning becomes difficult. Responses were also recorded on a five-point Likert-type scale from 1 = strongly disagree to 5 = strongly agree, and the scale score was calculated as the mean of valid items.

Because the study was conducted in a specific ethnic vocational context, all survey items were reviewed for linguistic clarity and contextual appropriateness before baseline administration. The review process included consultation with vocational school teachers, ethnic education instructors, and school social workers familiar with the Zhuang student population. Items were checked for semantic clarity, cultural appropriateness, and consistency with local school terminology. No item was intended to measure ethnic identity as a fixed personal trait; rather, the survey focused on school connection, learning confidence, and dropout-related experiences. Internal consistency reliability for the belonging and self-efficacy scales was examined in the analytic sample and is reported in Section 4 together with descriptive statistics. Cronbach’s alpha coefficients, item-total correlations, and scale means and standard deviations were reported to address measurement reliability in the present sample. Where applicable, confirmatory or exploratory checks of dimensional consistency were also used to support the use of mean scale scores.

### 3.5. Intervention Protocol and Collaborative Mechanism

The intervention consisted of two coordinated components, a school social work module and an ethnic education module, implemented within a school-level collaborative mechanism. The purpose of this structure was not simply to deliver multiple services at the same time, but to create an operational system in which risk identification, support planning, implementation, monitoring, and adjustment were linked across actors and time points. This design was intended to address dropout-related risk as an accumulated process rather than as a single behavioral event.

The school social work module followed a tiered support model. Students classified as lower risk received universal or light-touch support focused on study habits, attendance awareness, and basic adjustment guidance. Students identified as medium risk participated in structured group sessions addressing emotion regulation, conflict management, peer interaction, and short-term goal setting. Students identified as high risk received case management, including scheduled individual sessions, attendance planning, family contact, home visits where feasible, and referral or resource linkage when crisis or acute practical difficulties were identified. Risk stratification was updated during monthly review cycles using available attendance, discipline, and academic information. Risk level was determined from the same administrative and survey-based information used in the dropout risk monitoring system, but service allocation decisions were made by school social workers and homeroom teachers before outcome analysis to avoid post hoc classification.

The ethnic education module emphasized culturally responsive pedagogy and meaning construction. It linked Zhuang cultural resources, local identity recognition, and vocational learning in order to strengthen students’ perception that school participation was relevant to who they were and to what their future development could become. In practice, this module included culturally responsive lessons, identity-affirming activities, and learning support intended to reduce alienation associated with cultural mismatch, language-related discomfort, or weak perceived relevance of schooling. The module did not treat ethnic culture as an isolated symbolic activity; instead, it connected local cultural knowledge with vocational learning, peer participation, and future-oriented educational meaning.

The collaborative mechanism connected these two modules through biweekly case review and planning meetings. Participants in these meetings typically included social workers, homeroom teachers, ethnic education instructors, and relevant student affairs staff. The meetings were used to identify emerging risk indicators, review service progress, revise individual support priorities, and coordinate school–family communication. Shared support plans were developed for students requiring more intensive intervention, with explicit goals, responsible personnel, service intensity, and review timelines. Home–school–community coordination was used where necessary to improve family support and connect students or guardians with relevant local resources. This coordination mechanism was central to the intervention model because it allowed student risk information, family feedback, and classroom observations to be reviewed together rather than handled separately by different school actors.

Implementation fidelity was monitored through predefined indicators, including completion of screening cycles, case note completeness, home-visit completion, session attendance, lesson delivery checklists, participation rates, and documentation of joint case conferences. These indicators were used to document whether the intervention was delivered in a manner broadly consistent with the planned model and to support interpretation of outcome variation across groups and schools. Fidelity indicators were summarized at the class and intervention-group levels. They were not used to redefine group assignment in the primary intention-to-treat analysis, but they were used descriptively to assess whether the intervention was delivered with sufficient coverage and procedural consistency, Data are shown in Table 3.

### 3.6. Data Collection Procedures

Data collection was organized around the three formal study waves. At T0, prior to intervention implementation, the research team administered standardized student questionnaires in classroom settings and extracted administrative records from the previous semester, including attendance, disciplinary records, and academic indicators. At T1, immediately after the intervention period, the same survey measures were re-administered and administrative data generated during the intervention window were extracted. At T2, follow-up data were collected to evaluate retention status, attendance trajectories, chronic absenteeism, and the short-term maintenance of changes in school belonging and academic self-efficacy. The same variable definitions and extraction templates were used across all waves to maintain measurement consistency over time.

To reduce measurement error, all questionnaires were administered under standardized instructions using anonymous study codes. Administrative data were extracted by designated school staff according to predefined variable templates and were checked twice for consistency before analysis. Process documentation was maintained throughout the intervention period by social workers, teachers, and research staff, including service frequency, activity participation, family contact, and case conference records. Monthly review checks were conducted to improve consistency across schools. Any discrepancies between administrative records and class-level logs were reviewed with school staff before data locking. After data cleaning, identifying information was removed, and the final analytic dataset used anonymous study IDs only.

The qualitative component used semi-structured interviews with purposively selected participants representing different levels of risk exposure and intervention participation. Interviewees included students, guardians, homeroom teachers, ethnic education instructors, and school social workers. Interviews focused on perceived dropout pressures, support experiences, family–school interaction, cultural relevance of the intervention, and perceived changes in school connection and learning confidence. These materials were not used as anecdotal illustration alone; rather, they were analyzed systematically to support interpretation of mechanism pathways, implementation feasibility, and contextual variability in the quantitative findings. Integration of qualitative and quantitative evidence occurred mainly during interpretation. For example, interview themes concerning family communication, teacher recognition, and cultural relevance were compared with quantitative patterns in school belonging, academic self-efficacy, and subgroup responsiveness. This approach strengthened contextual interpretation, although the qualitative component remained complementary rather than fully equal in inferential weight.

### 3.7. Data Analysis Plan

Quantitative analyses adhered to an intention-to-treat (ITT) approach, ensuring all students were analyzed according to their original group allocation irrespective of the intervention dosage received. This approach was adopted to preserve comparability between groups and to avoid overstating the intervention effect by restricting analyses only to highly compliant participants. Descriptive analyses were first conducted to summarize baseline characteristics, attrition, intervention exposure, and distributional properties of the main variables. Baseline equivalence was assessed using *t* tests, non-parametric tests, or chi-square tests according to variable type and distribution.

Continuous outcomes—namely the dropout risk index, school belonging, and academic self-efficacy—were analyzed using linear mixed-effects models. These models incorporated fixed effects for group, time, and their interaction, alongside random effects to account for class-level clustering. The group × time coefficient was treated as the primary estimate of intervention-associated change. For each model, unstandardized coefficients, standard errors, exact *p*-values, 95% confidence intervals, and standardized effect sizes were reported. Model diagnostics included inspection of residual distributions and influential observations. Where applicable, model fit indices, including information criteria, were also reported to support transparency.

For binary outcomes, including chronic absenteeism and retention, generalized linear mixed models or clustered regression models with robust standard errors were used, depending on model convergence and distributional characteristics. The primary parameter of interest was again the group × time interaction, which captured whether outcome trajectories differed between the intervention and comparison groups across the study period. For binary models, estimates were reported as log-odds coefficients and, where helpful for interpretation, transformed into odds ratios with 95% confidence intervals. Exact *p*-values and model fit indicators were reported when available. Marginal predicted probabilities were used to aid interpretation of chronic absenteeism and retention differences across time.

Mediation analysis was conducted using a parallel mediation framework in which school belonging and academic self-efficacy were modeled as simultaneous mediators linking intervention exposure to dropout-related outcomes. Indirect effects were estimated separately for each mediator and then summarized as total indirect effects. Confidence intervals for indirect effects were obtained using bootstrap resampling procedures. Given the quasi-experimental nature of the design, mediation findings were interpreted cautiously as evidence of plausible explanatory pathways rather than definitive causal transmission. The mediation models reported direct effects, mediator-specific indirect effects, total indirect effects, total effects, bootstrap 95% confidence intervals, standard errors, and exact *p*-values where applicable.

Moderation and subgroup analyses were conducted to examine whether intervention-associated differences varied by baseline risk level, family support, and left-behind experience. Interaction terms were estimated rather than relying only on subgroup-specific descriptive comparisons. For each moderation model, the interaction coefficient, standard error, exact *p*-value, 95% confidence interval, and subgroup-specific marginal estimates were reported. These analyses were considered exploratory because the study was not primarily powered for fine-grained moderation testing.

Missing data were first examined to identify the likely missingness pattern. Depending on the results of this assessment, multiple imputation or full-information maximum likelihood estimation was used for the primary models. Sensitivity analyses compared results from the main analytic sample with alternative handling strategies for incomplete follow-up data. Additional sensitivity checks also examined whether the main findings were materially altered after adjustment for baseline covariates and school or class clustering. Further robustness checks included: (1) complete-case analysis versus imputed or likelihood-based analysis; (2) models with and without baseline covariates; (3) models using class-level clustered standard errors; (4) alternative specifications of the dropout risk index, including equal-weight and empirically standardized versions; and (5) exclusion of students with incomplete process exposure records. These checks were used to evaluate whether the main conclusions depended on a single modeling or scoring decision.

Qualitative data were analyzed using thematic analysis. Coding proceeded from initial open coding to focused theme development, with particular attention to intervention acceptability, relational support, culturally responsive experience, family involvement, and perceived changes in school engagement. Where possible, coding was cross-checked between researchers to improve interpretive consistency. Qualitative findings were then compared with the quantitative results to clarify whether observed statistical patterns were consistent with participants’ accounts of school connection, family communication, cultural recognition, and learning confidence. This procedure was used to strengthen interpretation rather than to claim independent qualitative generalizability.

## 4. Results

### 4.1. Participant Flow and Intervention Exposure

A total of 457 students were enrolled at baseline, including 230 in the intervention group and 227 in the comparison group. During follow-up, 22 students were not retained in the final analytic sample, primarily because of school transfer, withdrawal, or incomplete follow-up data. The final analytic sample therefore consisted of 435 students, including 218 in the intervention group and 217 in the comparison group. The attrition rate was 5.2% in the intervention group and 4.4% in the comparison group. The difference in attrition between groups was not statistically significant, χ^2^ = 0.16, *p* = 0.689, suggesting that follow-up loss was limited and broadly balanced across conditions.

Implementation records indicated that the intervention achieved relatively high coverage. Among students in the intervention group, 86.2% received at least one individual social work service, 71.6% participated in at least one group-based activity, and 78.4% of students classified as high risk completed at least one home-visit-based family contact. For the ethnic education component, 83.9% of intervention participants completed the planned courses or activities. The collaborative mechanism was implemented with acceptable regularity: joint review meetings were conducted as scheduled, meeting-minutes completeness reached 90.5%, and most high-need cases had shared support plans with periodic adjustment.

The mean number of documented social work contacts among intervention students was 3.42 (SD = 1.36), and the mean number of ethnic education activities attended was 4.18 (SD = 1.21). Among high-risk students, the mean number of individualized case-management contacts was 4.76 (SD = 1.44). These process indicators were not used to redefine group assignment in the intention-to-treat analysis, but they indicate that the intervention was delivered with sufficient procedural consistency to support interpretation of the outcome models. At the same time, some variation in actual participation remained, which should be considered when interpreting the magnitude of between-group differences.

### 4.2. Measurement Properties and Composite Dropout Risk Index

Before testing the main hypotheses, the reliability and metric properties of the principal survey-based and composite measures were examined. The school belonging scale showed good internal consistency across the three waves, with Cronbach’s α = 0.86 at T0, 0.88 at T1, and 0.87 at T2. Corrected item-total correlations ranged from 0.52 to 0.74. The academic self-efficacy scale also demonstrated acceptable to good internal consistency, with Cronbach’s α = 0.84 at T0, 0.86 at T1, and 0.86 at T2, and corrected item-total correlations ranging from 0.49 to 0.71. These results supported the use of mean scale scores for both mechanism variables.

The composite dropout risk index was constructed from four standardized domains: attendance burden, disciplinary risk, academic warning signals, and self-reported withdrawal tendency. Each domain contributed 25% to the final index. The inter-domain correlations were positive and moderate, ranging from r = 0.24 to r = 0.46, indicating that the four components captured related but non-identical aspects of dropout-related vulnerability. The internal coherence of the four-domain composite was acceptable for a multidimensional risk index, with α = 0.72 at T0. The equal-weighted index was highly correlated with an alternative principal-component-weighted specification, r = 0.96, suggesting that the substantive results were unlikely to depend on the selected weighting rule.

At baseline, the composite index was approximately standardized around zero in both groups, with higher scores indicating greater dropout-related risk. No substantial floor or ceiling effect was observed. The distribution was slightly right-skewed, as expected for a risk measure in a school-based sample, but inspection of residuals from the primary mixed model did not indicate serious violation of model assumptions. These findings supported the use of the composite index as a continuous outcome in the main longitudinal analysis.

### 4.3. Primary Outcomes

The descriptive outcome patterns across T0, T1, and T2 are presented in Table 4. At baseline, the intervention and comparison groups were similar on the main outcome indicators. Over time, the intervention group showed a more favorable pattern on the dropout risk index, chronic absenteeism, and retention than the comparison group. For the dropout risk index, the intervention group changed from a baseline mean (SD) of 0.00 (1.00) to −0.32 (0.92) at T1 and −0.55 (0.88) at T2. In the comparison group, the corresponding values were 0.01 (1.01), −0.10 (0.97), and −0.18 (0.95). By T2, the unadjusted between-group difference was −0.37. For chronic absenteeism, the intervention group decreased from 22.9% at baseline to 14.7% at T1 and 11.0% at T2, whereas the comparison group changed from 21.7% at baseline to 18.9% at T1 and 18.0% at T2. The between-group difference at T2 was −7.0 percentage points. For retention, both groups remained high overall, but the intervention group showed a modest advantage at follow-up. At T1, retention was 98.2% in the intervention group and 96.8% in the comparison group. At T2, the corresponding figures were 97.7% and 94.9%, yielding an unadjusted between-group difference of 2.8 percentage points.

The adjusted model results are reported in Table 5. In the adjusted linear mixed-effects model, the group × time interaction for the dropout risk index was statistically significant at T1, β = −0.21, SE = 0.07, 95% CI [−0.35, −0.07], *p* = 0.003, standardized effect size d = −0.22, and became stronger at T2, β = −0.37, SE = 0.08, 95% CI [−0.52, −0.22], *p* < 0.001, d = −0.40. This indicates that the intervention group showed a larger reduction in dropout-related risk over time after adjustment for baseline covariates and class-level clustering. For chronic absenteeism, the intervention-associated difference at T1 was in the expected direction but did not reach conventional statistical significance, log-odds β = −0.34, SE = 0.24, OR = 0.71, 95% CI [0.44, 1.15], *p* = 0.162. At T2, the group × time effect was statistically significant, log-odds β = −0.58, SE = 0.25, OR = 0.56, 95% CI [0.34, 0.91], *p* = 0.020. The marginal predicted probability of chronic absenteeism at T2 was 11.6% in the intervention group and 18.5% in the comparison group. For retention, the adjusted binary model showed a positive but statistically non-significant intervention-associated difference at T2, log-odds β = 0.53, SE = 0.39, OR = 1.70, 95% CI [0.79, 3.67], *p* = 0.174. Given the high retention rate in both groups and the small number of withdrawal events, this estimate should be interpreted cautiously.

Overall, the primary outcome models supported H1 most clearly for the dropout risk index and chronic absenteeism. The evidence for retention was directionally consistent but weaker, probably because formal non-retention was relatively infrequent during the short follow-up period. These findings indicate intervention-associated differences in dropout-related outcomes, although the quasi-experimental design does not permit strong causal interpretation.

### 4.4. Secondary Outcomes and Mechanism Variables

The two proposed mechanism variables also showed more favorable change in the intervention group than in the comparison group, as shown descriptively in Table 4 and analytically in Table 5. Detailed descriptive trajectories and adjusted model estimates for the mechanism variables are consolidated in Table 4 and Table 5, respectively. Specifically, school belonging in the intervention group exhibited a steady increase across the three waves, resulting in a notable unadjusted between-group advantage at T2. The adjusted group × time coefficient was β = 0.27, SE = 0.05, 95% CI [0.17, 0.37], *p* < 0.001 at T1, and β = 0.33, SE = 0.06, 95% CI [0.21, 0.45], *p* < 0.001 at T2. The standardized effect size at T2 was moderate, d = 0.64. These findings support H2.

Academic self-efficacy followed a similar pattern. The intervention group increased from 3.02 (0.56) at baseline to 3.35 (0.53) at T1 and 3.44 (0.52) at T2, whereas the comparison group changed from 3.04 (0.55) to 3.12 (0.54) and 3.16 (0.55), respectively. The unadjusted between-group difference at T2 was 0.28. In the adjusted model, the group × time coefficient was β = 0.25, SE = 0.05, 95% CI [0.15, 0.35], *p* < 0.001 at T1, and β = 0.30, SE = 0.06, 95% CI [0.18, 0.42], *p* < 0.001 at T2. The standardized effect size at T2 was d = 0.56. These findings support H3.

Although not a primary outcome, disciplinary incidents also declined in both groups, with a somewhat larger reduction in the intervention group. The proportion of students with any disciplinary incident fell from 15.6% to 8.3% in the intervention group and from 15.2% to 12.0% in the comparison group by T2, corresponding to a between-group difference of −3.7 percentage points. However, this difference was not statistically significant in the adjusted binary model, log-odds β = −0.36, SE = 0.27, OR = 0.70, 95% CI [0.41, 1.18], *p* = 0.183. Therefore, disciplinary incidents were treated as a supportive behavioral indicator rather than as a primary basis for inference.

### 4.5. Mediation Findings

The parallel mediation results are presented in Table 6. Parallel mediation analysis indicated that both school belonging and academic self-efficacy were associated with the relationship between intervention exposure and the dropout risk index. The indirect effect through school belonging was −0.12, SE = 0.03, 95% CI [−0.19, −0.06], *p* < 0.001, and the indirect effect through academic self-efficacy was −0.08, SE = 0.03, 95% CI [−0.14, −0.03], *p* = 0.004. The total indirect effect was −0.20, SE = 0.04, 95% CI [−0.28, −0.12], *p* < 0.001. The remaining direct effect was −0.17, SE = 0.06, 95% CI [−0.30, −0.05], *p* = 0.006. The total effect was −0.37, SE = 0.08, 95% CI [−0.52, −0.22], *p* < 0.001. Approximately 54.1% of the total association was statistically accounted for by the two parallel mediators.

These results are consistent with the hypothesized role of school belonging and academic self-efficacy as parallel explanatory pathways. The indirect pathway through school belonging appeared somewhat larger than the pathway through academic self-efficacy, suggesting that improvement in students’ relational connection to school may have been particularly relevant to the observed reduction in dropout-related risk. However, because the mediation model was estimated within a quasi-experimental design, these results should be interpreted as evidence of plausible mechanisms rather than definitive proof of causal transmission.

Mediation analyses for chronic absenteeism and retention showed directionally similar but less stable patterns. For chronic absenteeism, the total indirect pathway through the two mediators was negative but smaller in magnitude, β = −0.09, SE = 0.04, 95% CI [−0.18, −0.02], *p* = 0.031. For retention, the indirect pathway was positive but imprecisely estimated, β = 0.05, SE = 0.04, 95% CI [−0.02, 0.14], *p* = 0.147. These results suggest that school belonging and academic self-efficacy were most clearly linked to the continuous dropout risk index, while evidence for mediation of formal retention was weaker because retention events were relatively rare. Overall, the mediation results are broadly consistent with H4, H5, and H6.

### 4.6. Moderation, Subgroup Findings, and Sensitivity Analyses

The moderation and sensitivity analysis results are summarized in Table 7. Subgroup and moderation analyses suggested that intervention-associated differences were not uniform across all students. The intervention appeared more strongly associated with reduced dropout-related risk among students who entered the study with higher baseline risk. In the high-baseline-risk subgroup, the estimated difference in the dropout risk index was −0.52, SE = 0.11, 95% CI [−0.73, −0.31], *p* < 0.001, compared with −0.21, SE = 0.10, 95% CI [−0.40, −0.02], *p* = 0.031 in the lower-risk subgroup. The formal interaction between intervention status and baseline risk level was statistically significant, β = −0.31, SE = 0.14, 95% CI [−0.58, −0.04], *p* = 0.026.

Family support and left-behind experience also showed differentiated patterns. Among students with lower baseline family support, the estimated difference in chronic absenteeism was −0.10, SE = 0.04, 95% CI [−0.18, −0.02], *p* = 0.018, whereas the corresponding estimate for students with higher family support was −0.04, SE = 0.03, 95% CI [−0.10, 0.02], *p* = 0.196. The interaction term for family support was in the expected direction but did not reach conventional statistical significance, β = −0.06, SE = 0.04, 95% CI [−0.14, 0.02], *p* = 0.128.

For school belonging, the intervention-associated gain was 0.34, SE = 0.08, 95% CI [0.18, 0.50], *p* < 0.001 in the left-behind subgroup and 0.22, SE = 0.07, 95% CI [0.08, 0.36], *p* = 0.002 in the non-left-behind subgroup. The interaction term for left-behind experience was positive but only marginally significant, β = 0.12, SE = 0.07, 95% CI [−0.01, 0.25], *p* = 0.071.

Sensitivity analyses were conducted to examine the robustness of the main findings. First, complete-case analyses produced estimates similar to those obtained from the main adjusted models. For the dropout risk index at T2, the complete-case estimate was β = −0.35, SE = 0.08, 95% CI [−0.51, −0.20], *p* < 0.001. Second, models without baseline covariates yielded a comparable estimate, β = −0.39, SE = 0.08, 95% CI [−0.55, −0.24], *p* < 0.001. Third, using clustered robust standard errors instead of random class effects did not materially change the inference, β = −0.36, SE = 0.09, 95% CI [−0.53, −0.19], *p* < 0.001. Fourth, the alternative principal-component-weighted dropout risk index produced a similar group × time estimate, β = −0.34, SE = 0.08, 95% CI [−0.50, −0.18], *p* < 0.001. Fifth, excluding students with incomplete process exposure records did not reverse the direction or significance of the main findings, β = −0.38, SE = 0.09, 95% CI [−0.56, −0.21], *p* < 0.001.

These findings suggest that the intervention may have been particularly relevant for students facing greater accumulated vulnerability, including those with elevated baseline risk, weaker family support, or left-behind experience. At the same time, these subgroup analyses should be interpreted cautiously because the study was not primarily powered for fine-grained moderation testing and residual confounding cannot be fully excluded. The strongest and most robust evidence was observed for the dropout risk index and the two proposed mechanism variables, whereas retention and some subgroup effects were directionally consistent but less precise.

## 5. Discussion

This study examined whether a collaborative intervention integrating school social work and ethnic education was associated with more favorable dropout-related outcomes among male students in vocational schools in central Guangxi. Across the three-wave observation period, the intervention group showed a larger reduction in the dropout risk index and a lower probability of chronic absenteeism than the comparison group, while the difference in retention was positive but less precise. The intervention group also reported stronger school belonging and academic self-efficacy over time. These findings should be interpreted as adjusted associations within a quasi-experimental school-based design rather than as definitive causal effects. Even with this caution, the pattern suggests that a culturally situated and relationally coordinated intervention may offer a useful approach for addressing dropout-related vulnerability in ethnic minority vocational education settings (Peguero & Shaffer, 2015). These findings align with broader international evidence demonstrating that culturally responsive practices and targeted socio-emotional support are critical for retaining marginalized youth in vocational pathways, akin to challenges faced by immigrant-background apprentices in European VET systems or minoritized students in the United States (Blommaert et al., 2020). By contextualizing this within China, the present study highlights a cross-cultural imperative: vocational retention requires simultaneously addressing structural skill deficits and cultural alienation.

One contribution of the present study is that it shifts the analytical focus away from dropout as a single terminal event and toward dropout-related risk as a dynamic and potentially interruptible process. The results are consistent with the view that absenteeism, disengagement, weak school connection, and reduced confidence in learning do not operate in isolation. Rather, they appear to cluster and accumulate over time (Qian, 2013). This interpretation is supported by the stronger and more stable findings for the continuous dropout risk index than for formal retention. Because formal withdrawal events were infrequent during the short follow-up period, the composite risk index proved more sensitive in capturing early shifts in disengagement, such as attendance burdens and academic warnings. Consequently, the collaborative intervention’s effectiveness likely stems not from merely increasing service volume, but from deliberately integrating risk identification, relational support, and culturally responsive meaning-making (Shi et al., 2015).

The mediation findings provide further support for the relevance of school belonging and academic self-efficacy as protective processes. The indirect pathway through school belonging was somewhat larger than the pathway through academic self-efficacy, suggesting that students’ perceived recognition, acceptance, and connection to school may have been particularly relevant to the observed reduction in dropout-related risk (Sulimani-Aidan & Melkman, 2022). This is important in vocational education settings where persistence is often shaped not only by academic capacity, but also by whether students feel that schooling remains socially and personally worthwhile (Tanner-Smith & Wilson, 2013). At the same time, the indirect pathway through academic self-efficacy indicates that confidence in handling learning and vocational tasks may also contribute to lower dropout-related vulnerability. The novelty of the present study lies in modeling how these components operate through identifiable protective processes within a culturally situated vocational context. By demonstrating the efficacy of identity affirmation, this study resonates with international calls for “culturally sustaining” practices (Paris & Alim, 2017) and underscores the importance of aligning institutional support with students’ socio-personal agency in vocational pathways (Billett, 2014). School social work appeared to support risk monitoring, case follow-up, and family communication, whereas ethnic education appeared to strengthen school meaning, cultural recognition, and belonging. These two mechanisms should not be treated as competing explanations; they suggest that relational connection and competence belief may work together in sustaining school participation (World Bank Group, 2018).

The subgroup findings also merit attention. Intervention-associated differences were larger among students with higher baseline risk and among some students facing weaker family support or left-behind experience. This pattern is substantively plausible. Students who enter vocational schooling with accumulated attendance problems, unstable supervision, or greater psychosocial strain may have more to gain from structured follow-up, family contact, and stronger school connection (Yi et al., 2015). However, the moderation results should be read with restraint. The interaction for baseline risk was statistically supported, whereas the interaction patterns for family support and left-behind experience were weaker or marginal. These findings are therefore better understood as signals of possible heterogeneity rather than as firm evidence that the intervention works differently for clearly defined subgroups. Future studies with larger clustered samples and prespecified moderation hypotheses are needed before subgroup-specific implementation claims can be made.

Despite its contributions, several methodological constraints warrant caution. First, the non-randomized, quasi-experimental design precludes robust causal inferences; the observed outcomes represent adjusted associations rather than definitive effects. Second, the follow-up duration was insufficient to evaluate long-term educational trajectories, limiting our ability to estimate sustained retention beyond the immediate intervention cycle. Third, the focus on male students in specific Zhuang vocational contexts inherently constrains the external validity of the findings to other genders or diverse ethnic settings. Finally, the qualitative component functioned as a complementary explanatory strand with less inferential weight than the quantitative analysis, restricting the depth of independent triangulation (Pusztai et al., 2025). This limits the extent to which qualitative evidence can be used to independently validate the quantitative findings, even though it helped clarify how students, families, teachers, and social workers experienced the intervention process.

The cultural specificity of the study is both a strength and a boundary condition. The intervention was developed in vocational schools serving Zhuang communities in central Guangxi, and its relevance was closely tied to local identity, school structure, and family–school relations. For that reason, the findings should not be generalized mechanically to all vocational settings, all ethnic minority regions, or female student populations. The more transferable contribution is the underlying intervention logic: dropout prevention in culturally diverse vocational environments may require coordination between relational support, family engagement, culturally meaningful pedagogy, and continuous risk monitoring. This principle aligns with global educational equity initiatives, such as the European “Second Chance” vocational frameworks and the US-based “Culturally Sustaining Pedagogies,” which emphasize that retention for minoritized youth depends on bridging the gap between home culture and institutional expectations (Wang, 2022). By integrating these international paradigms, the present study demonstrates that while the concrete content of ethnic education must be localized, the underlying mechanism—reciprocal relational support combined with identity affirmation—remains a cross-cultural imperative for vocational retention.

From a practical standpoint, the present study suggests three implications. First, dropout prevention in vocational schools may benefit from shifting from reactive management toward earlier, tiered, and data-informed support. Second, ethnic education may be more useful when it moves beyond symbolic or activity-based representation and becomes connected to learning meaning, school participation, and future development. Third, school-level coordination matters. Without regular communication, shared planning, and procedural continuity across teachers, social workers, and family-facing staff, even well-designed supports may remain fragmented. These implications should be viewed as provisional and context-sensitive. The present evidence supports the feasibility and promise of a coordinated model, but it does not justify universal implementation claims without further replication under stronger research designs (Krötz & Deutscher, 2022).

More broadly, the study contributes to the literature by showing how dropout prevention can be examined through protective processes rather than through program labels alone. Its scientific contribution is therefore not simply that it combines two intervention components, but that it specifies a culturally situated mechanism model in which school belonging and academic self-efficacy help explain why a collaborative intervention may be associated with lower dropout-related risk. Future research could strengthen this line of work by extending follow-up duration, incorporating richer controls for school climate and mental health, using more explicit trajectory modeling, and developing a more fully integrated mixed-methods design with stronger qualitative triangulation. Randomized or matched quasi-experimental designs across a larger number of schools would also help clarify whether the associations observed here remain stable when selection bias, class-level clustering, and school-level variation are more fully addressed.

## 6. Conclusions

This study examined a collaborative intervention integrating school social work and ethnic education for male students in vocational schools in central Guangxi. The findings indicate that, relative to usual school support, the intervention group showed more favorable patterns in dropout risk, chronic absenteeism, retention, school belonging, and academic self-efficacy across the study period. The results further suggest that school belonging and academic self-efficacy may function as important explanatory pathways linking intervention exposure to dropout-related outcomes.

Given the lack of individual randomization and the context-specific sample, these findings represent adjusted associations rather than experimental proof. Nevertheless, they offer a mechanistic model for multi-level retention governance. Specifically, the study demonstrates that dropout prevention in ethnically diverse vocational settings benefits substantially from integrating tiered relational support, family engagement, and culturally responsive educational practices. Rather than relying only on post hoc management of visible absenteeism, schools may need more coordinated systems that address both the behavioral and motivational dimensions of disengagement.

In this sense, the present study offers a context-sensitive model for strengthening school retention efforts in vocational education, while also indicating the need for further research using longer follow-up, stronger causal designs, and broader contextual measures.

## Figures and Tables

**Table 1:** Participant Characteristics and Baseline Comparability.

Characteristic	Intervention Group n Equals 230	Control Group n Equals 227	*p* Value
Age in years mean SD	16.41 (0.78)	16.39 (0.80)	0.74
Grade 10 n percent	96 (41.7)	98 (43.2)	0.77
Grade 11 n percent	82 (35.7)	78 (34.4)	0.79
Grade 12 n percent	52 (22.6)	51 (22.5)	0.98
Zhuang ethnicity n percent	162 (70.4)	156 (68.7)	0.68
Boarding student n percent	178 (77.4)	172 (75.8)	0.67
Left behind experience n percent	104 (45.2)	99 (43.6)	0.73
Baseline semester absence days median IQR	6 3 to 11	6 3 to 10	0.61
Chronic absenteeism at baseline n percent	54 (23.5)	49 (21.6)	0.63
Baseline academic score mean SD	69.8 (9.6)	70.4 (9.4)	0.48
Any disciplinary record at baseline n percent	38 (16.5)	35 (15.4)	0.75
Perceived family economic pressure high n percent	86 (37.4)	81 (35.7)	0.7

**Table 2:** Measures and Data Sources.

Construct	Operational Indicator	Data Source	Time Points
Dropout risk	Standardized composite index combining attendance burden, disciplinary risk, academic warning signals, and self-reported withdrawal tendency; each domain contributed 25% to the final score	Administrative records plus student survey	T0, T1, T2
Chronic absenteeism	Binary indicator of absence proportion reaching or exceeding the school administrative warning threshold	Attendance system	T0, T1, T2
Retention	Enrollment status without formal withdrawal or transfer out	Student status registry	T1, T2
Attendance	Absence days and tardiness counts	Attendance system	T0, T1, T2
Disciplinary incidents	Any incident and incident counts	Student affairs records	T0, T1, T2
School belonging	Mean score of adapted school connectedness items; five-point Likert scale; higher scores indicate stronger belonging	Student survey	T0, T1, T2
Academic self-efficacy	Mean score of adapted academic self-efficacy items; five-point Likert scale; higher scores indicate stronger efficacy beliefs	Student survey	T0, T1, T2
Baseline covariates	Age, grade, ethnicity, boarding status, left-behind experience, baseline absence burden, baseline academic score, disciplinary history, and perceived family economic pressure	Student survey plus school registry	T0
Intervention exposure	Class-level intervention assignment and implementation records, including service participation and activity completion	Allocation records and process documentation	T1, T2
Implementation fidelity	Screening completion, case-note completeness, home-visit completion, session attendance, lesson delivery checklist score, participation rate, and case-conference documentation	Process records	During intervention

**Table 3:** Intervention Components and Fidelity Indicators.

Module	Component	Core Activities	Planned Dose	Provider	Fidelity Indicators
Social Work	Tiered risk screening	Monthly risk review using attendance, academic discipline	Once per month	School social worker team	Screening completed rate percent
Social Work	Case management for high risk	Individual sessions goal-setting, problem-solving attendance plan	Two sessions per month	Social worker	Case notes completeness rate percent
Social Work	Family engagement	Home visit caregiver communication training resource linkage	One visit per month for high risk	Social worker plus homeroom teacher when needed	Home visit completion rate percent
Social Work	Group program for medium risk	Emotion regulation conflict management peer norms and career planning	Six sessions per cycle	Social worker	Session attendance rate percent
Ethnic Education	Culturally responsive learning	Integrate Zhuang culture with career learning meaning making	Two lessons per month	Ethnic education teacher	Lesson delivery checklist score
Ethnic Education	Identity affirmation activities	Cultural pride, reflection, school belonging activities	One activity per month	Ethnic education teacher	Student participation rate percent
Collaboration	Joint case conference	Joint review shared plan adjustment referral decisions	Every two weeks	Social worker plus school staff	Meeting minutes completion rate percent

**Table 4:** Primary and Secondary Outcomes.

Outcome	Time	Intervention Group	Control Group	Between-Group Difference at T2
Dropout risk index (higher = worse)	T0	0.00 1.00	0.01 1.01	
	T1	−0.32 0.92	−0.10 0.97	
	T2	−0.55 0.88	−0.18 0.95	−0.37
Chronic absenteeism	T0	50 22.9	47 21.7	
	T1	32 14.7	41 18.9	
	T2	24 11.0	39 18.0	−7.0 percent
Retention	T1	214 98.2	210 96.8	
	T2	213 97.7	206 94.9	2.8 percent
School belonging (higher = better)	T0	3.10 0.54	3.12 0.55	
	T1	3.48 0.50	3.23 0.53	
	T2	3.56 0.49	3.25 0.54	0.31
Academic self-efficacy (higher = better)	T0	3.02 0.56	3.04 0.55	
	T1	3.35 0.53	3.12 0.54	
	T2	3.44 0.52	3.16 0.55	0.28
Any disciplinary incident	T0	34 15.6	33 15.2	
	T1	21 9.6	28 12.9	
	T2	18 8.3	26 12.0	−3.7 percent

**Table 5:** Adjusted Primary and Secondary Outcome Models.

Outcome	Model Parameter	β (Log-Odds)	SE	95% CI	*p* Value	Effect Size/OR
Dropout risk index	Group × T1	−0.21	0.07	−0.35 to −0.07	0.003	d = −0.22
Dropout risk index	Group × T2	−0.37	0.08	−0.52 to −0.22	<0.001	d = −0.40
Chronic absenteeism	Group × T1	−0.34	0.24	−0.78 to 0.14	0.162	OR = 0.71
Chronic absenteeism	Group × T2	−0.58	0.25	−1.08 to −0.09	0.020	OR = 0.56
Retention	Group × T2	0.53	0.39	−0.23 to 1.30	0.174	OR = 1.70
School belonging	Group × T1	0.27	0.05	0.17 to 0.37	<0.001	d = 0.52
School belonging	Group × T2	0.33	0.06	0.21 to 0.45	<0.001	d = 0.64
Academic self-efficacy	Group × T1	0.25	0.05	0.15 to 0.35	<0.001	d = 0.47
Academic self-efficacy	Group × T2	0.30	0.06	0.18 to 0.42	<0.001	d = 0.56
Any disciplinary incident	Group × T2	−0.36	0.27	−0.89 to 0.17	0.183	OR = 0.70

Note. Continuous outcomes were estimated using linear mixed-effects models. Binary outcomes were estimated using generalized mixed models or clustered regression models with robust standard errors. Models adjusted for baseline value where applicable, grade, boarding status, left-behind experience, baseline academic score, baseline absence burden, disciplinary history, and perceived family economic pressure. Class-level clustering was accounted for through random effects or clustered standard errors.

**Table 6:** Mediation and Moderation Results.

Pathway	Outcome	Effect Estimate	SE	95% CI	*p*-Value
Indirect effect via school belonging	Dropout risk index	−0.12	0.03	−0.19 to −0.06	<0.001
Indirect effect via academic self-efficacy	Dropout risk index	−0.08	0.03	−0.14 to −0.03	0.004
Total indirect effect	Dropout risk index	−0.20	0.04	−0.28 to −0.12	<0.001
Direct effect	Dropout risk index	−0.17	0.06	−0.30 to −0.05	0.006
Total effect	Dropout risk index	−0.37	0.08	−0.52 to −0.22	<0.001
Total indirect effect	Chronic absenteeism	−0.09	0.04	−0.18 to −0.02	0.031
Total indirect effect	Retention	0.05	0.04	−0.02 to 0.14	0.147

Note. Indirect effects were estimated using a parallel mediation model with school belonging and academic self-efficacy entered simultaneously. Confidence intervals were obtained using bootstrap resampling. Effects are interpreted as plausible explanatory pathways rather than causal mediation effects because of the quasi-experimental design.

**Table 7:** Moderation and Sensitivity Analysis Results.

Analysis	Outcome	Effect Estimate	SE	95% CI	*p* Value
High baseline risk subgroup	Dropout risk index	−0.52	0.11	−0.73 to −0.31	<0.001
Lower baseline risk subgroup	Dropout risk index	−0.21	0.10	−0.40 to −0.02	0.031
Intervention × high baseline risk	Dropout risk index	−0.31	0.14	−0.58 to −0.04	0.026
Lower family support subgroup	Chronic absenteeism	−0.10	0.04	−0.18 to −0.02	0.018
Higher family support subgroup	Chronic absenteeism	−0.04	0.03	−0.10 to 0.02	0.196
Intervention × lower family support	Chronic absenteeism	−0.06	0.04	−0.14 to 0.02	0.128
Left-behind subgroup	School belonging	0.34	0.08	0.18 to 0.50	<0.001
Non-left-behind subgroup	School belonging	0.22	0.07	0.08 to 0.36	0.002
Intervention × left-behind experience	School belonging	0.12	0.07	−0.01 to 0.25	0.071
Complete-case model	Dropout risk index	−0.35	0.08	−0.51 to −0.20	<0.001
Model without baseline covariates	Dropout risk index	−0.39	0.08	−0.55 to −0.24	<0.001
Clustered robust SE model	Dropout risk index	−0.36	0.09	−0.53 to −0.19	<0.001
PCA-weighted risk index	Dropout risk index	−0.34	0.08	−0.50 to −0.18	<0.001
Excluding incomplete process records	Dropout risk index	−0.38	0.09	−0.56 to −0.21	<0.001

## Data Availability

Data are included within the manuscript; further requests can be made directly to the corresponding author.
